# Predictors of first-line antiretroviral therapy failure amongst HIV-infected adult clients at Woldia Hospital, Northeast Ethiopia

**DOI:** 10.1371/journal.pone.0187694

**Published:** 2017-11-02

**Authors:** Yohannes Demissie Babo, Getahun Asres Alemie, Fasil Walelign Fentaye

**Affiliations:** 1 Management Science for Health, Bahirdar, Ethiopia; 2 Public Health Institute, College of Medicine & Health Sciences, University of Gondar, Gondar, Ethiopia; 3 Department of Public Health, College of Medicine & Health Sciences, Wollo University, Dessie, Ethiopia; University of Texas Health Science Center at San Antonio, UNITED STATES

## Abstract

**Background:**

Due to the limited availability of viral load testing for treatment outcome monitoring in resource limited settings, identifying predictive factors of antiretroviral treatment failure will help in selecting clients who will benefit most from the targeted use of viral load monitoring. Little is known about the predictors of treatment failure in the study area. This study was conducted to determine factors that predict first-line antiretroviral therapy failure among HIV-infected adult clients at Woldia Hospital, Northeast Ethiopia. For this study, antiretroviral therapy treatment failure was defined as the fulfillment of clinical and/or immunological criteria set by WHO.

**Methods:**

Case-control study was carried out from November to December 2014. Cases were adult clients who were on failing first line regimen and on active follow up while controls were those adult clients on a non-failing first-line regimen for 36 months and above and on active follow up. Data was entered in to Epi Info version 7 and was exported to SPSS version 20 for analysis. Binary logistic regression model was used to identify predictors of ART failure.

**Results:**

A total of 59 cases and 245 controls were included in the analysis. Sixty three percent of the participants were females and the median age at ART enrollment was 33 years (IQR; 28, 40). The median baseline CD_4_count was not significantly different among cases and controls (105 (IQR = 60–174)vs.131 (IQR = 72.5–189.0); p = 0.301). The median peak CD4 count in the failure group (230 (IQR = 123–387)) was significantly low compared to the non-failure group (463 (IQR = 348.5–577)) [p < 0.001]. High peak CD4count (AOR = 0.993; 95% CI 0.990, 0.996) and longer duration on ART (AOR = 0.923; 95% CI 0.893, 0.954) were protective of treatment failure. In addition stavudine based regimen (AOR = 3.47; 95% CI 1.343, 10.555), low baseline BMI (AOR = 2.75; 95% CI 1.012, 7.457), unemployment (AOR = 4.93; 95% CI 1.493, 16.305) and formal educational level (AOR = 5.15; 95% CI 1.534, 17.276) were independently significant predictors of treatment failure.

**Conclusions:**

In this setting low peak CD4count, shorter duration on first line ART, d4T based regimen, low baseline BMI, unemployment and formal educational level were significantly associated with increased treatment failure. Retaining patients on their initial first line regimen with appropriate follow up and improving their socioeconomic status through various livelihood initiatives should be strengthened.

## Introduction

Globally, an estimated 36.7 million people were living with Human Immunodeficiency Virus (HIV) in 2015. Sub-Saharan Africa remains most severely affected, with nearly 25.5 million people living with HIV and accounting for 69.5% of the people living with HIV worldwide [1]. Ethiopia is among the countries most affected by the HIV epidemic,with 793,700 people living with HIV in 2013 [2, 3].

The introduction of Anti-Retroviral Therapy (ART) was a critical turning point in the history of HIV disease and the gradual evolution of HIV infection into a chronic non-fatal condition. The primary goal of ART is to achieve and sustain maximal viral suppression [4, 5]. The global coverage for antiretroviral therapy reached 46%, in 2015, showing a remarkable increase (from 24% in 2010 to 54%) in the most affected areas, like eastern and southern Africa region. This pushes the number of people on treatment to 10.3 million [1,6].

In Ethiopia, fee based ART was launched in 2003 and subsequently in 2005 free ART program was initiated. Guidelines for starting ART have progressively shifted away from the initial threshold of ‘less than 200 CD_4_ cells/mm^3^’ in 2005 to a recommendation of ‘less than 350 CD_4_ cells/mm^3^’ in 2008. Currently, adults with WHO clinical stage III & IV irrespective of CD_4_ cell count or CD_4_ cell count less than 500 cell/mm^3^ or all TB/HIV co-infected and pregnant & lactating women irrespective of CD_4_ cell counts are eligible for ART. The basic first-line regimen contains 2 Nucleoside Reverse Transcriptase Inhibitors (NRTIs) as a backbone and 1 Non-nucleoside Reverse Transcriptase Inhibitors (NNRTIs). The choice of second-line regimen depends on the Anti-retroviral drugs (ARVs) used for first-line and that retain activity against the virus. Generally, one or more drug from a new class, usually Protease Inhibitors (PIs), is included [2, 5, 7].

Antiretroviral treatment (ART) failure is associated with virologic failure, immunologic failure, and/or clinical failure. Virological failure is said to be occurred when plasma viral load become above 1000 copies/ml in two consecutive measurements within a three month interval with adherence support after at least six months of using ART. Whereas, immunological failure is defined as a fall in CD_4_ cell count to baseline (or below) or a 50% reduction from on treatment peak value or presence of persistent CD_4_ cell count below 100 cells/mm^3^. In addition, clinical failure is the occurrence of new or recurrent WHO stage 4 or some stage 3 conditions [5, 7].

According to the 2011 Ethiopian Demographic and Health Survey, the prevalence of HIV in Amhara region is 1.6%, which is slightly higher than the national prevalence. The HIV situation in the region is one of the worst in the country, especially in the urban areas,having one third of People Living with HIV/AIDS (PLWHA) and those in need of ART found. Woldia town is a transport corridor in the Eastern part of Amhara and is hot spot area for HIV infection [3, 8]. In the majority of cases, untreated HIV infection progressively weakens the host immunity leading to Acquired Immunodeficiency Syndrome (AIDS) and non-AIDS defining illness and eventual death. The introduction of ART has brought a dramatic change in AIDS related deaths, illness, a drop in new infections including mother to child transmission and improvement in the quality of life of HIV infected peoples [5].

Meeting universal access rapidly and maintaining patients on appropriate treatment regimen are crucial to the long-term success of ART program. Patients are required to consistently adhere to the lifelong therapy to minimize the development of drug resistance [6]. Despite impressive results of the rapid scale-up of ART in Ethiopia, reduced patient retention on appropriate first-line drug overtime and increasing lost-to-follow up, which result in emergence of drug resistance and treatment failure, are becoming a challenge. Findings from prior HIV drug resistance early warning indicators surveys revealed that, among the health facilities 44% in 2008, 22% in 2009 and 20% in 2010 did not meet WHO recommendations of having ≥70% retention on first line ART at 12 months [9].

Monitoring individuals receiving ART is important to ensure successful treatment, identify adherence problems and determine regimen switch in case of treatment failure. Viral load monitoring is a more sensitive and an early indicator of treatment failure. However, most ART programs in resource limited countries didn’t have access to viral load testing. This limited use of viral load monitoring has been identified as a key reason for the lower rate of switching to second-line regimen than expected [10].

Although viral load monitoring is the gold standard method to diagnose ART failure, it is not accessible in resource-limited settings like Ethiopia. Hence, the diagnosis and monitoring of first-line treatment failure and the decision to initiate second-line treatment are largely based on clinical and immunologic assessment of patients. Clinico-immunologic determination of ART failure, in the absence of viral load, are associated with unnecessary switches to second-line in the absence of virologic failure and the prolongation of a failing regimen and a late switch to second-line. Late switches of first-line regimens, which contain NNRTIs, are associated with accumulation of mutation and lead to cross-resistance to other NNRTI drug that might be used as a second-line option [11, 12, 13].

In Ethiopia, according to FMOH report in January 2013, approximately 1.5% patients currently on ART are receiving second-line treatment [14]. The rate of switch to second-line regimen remained low pertaining to the maturity of the ART program [15]. A study conducted at Debremarkos hospital, Northwest part of Ethiopia, indicated a 21% of immunologic failure and with more than half of the immunological failure occurring in the first 12 months of ART initiation [16].

Recent surveys by WHO indicated that there are increased signs of both transmitted and acquired HIV Drug Resistance (HIVDR) in areas where ART program was expanded. Little is known about factors that predict treatment failure in Ethiopia, in general, and in the study area, in particular. Identifying such factors has programmatic importance, as cost effective interventions can be made at program level to maintain the efficacy of standard regimens [17, 18].

### Monitoring of treatment response in patients on ART

Different studies indicated that immunologic criteria have low sensitivity to detect virologic failure as they caused a large number of patients to be misdiagnosed and continued to take a failing regimen. In addition, their specificity & predictive values are also low, leading to unnecessary switches with increased cost for the expensive second-line drugs [19, 20, 21, 22].

Despite the limitation in the diagnostic capacity to detect treatment failure in sub-Saharan Africa, high viral suppression rate for substantial length of time and enhanced immunologic recovery has been documented. Patients with very low baseline CD_4_ cell counts (<50 cells/mm^3^) have equivalent or greater rate of immune recovery in the first year of ART compared to those with a higher baseline CD_4_ cell counts [23, 24, 25].

### Magnitude and factors associated with ART failure

Discordant virological and immunological responses are observed during ART suggesting a complex interaction between virological response and the CD_4_ cell count change [20, 23]. A 21% of immunologic failure was reported from a study at Debremarkos hospital, Ethiopia. From this study, having recurrent pneumonia infection at baseline, being unemployed, inability to work due to health problem, baseline CD_4_ cell count and body weight change were associated to immunological failure [16]. A similarly higher level of immunologic failure was found in the studies conducted in Mozambique and EuroSIDA. The study on fee based ART program in Mozambique showed that, the risk of failure was higher among patients with a high baseline CD_4_ cell count and low baseline viral load. The result reflected that these groups of patients might have low adherence level due to the lower risk of developing opportunistic infections [26, 27]. Contrary to these findings, a low rate of immunologic treatment failure was reported from a study in India [28].

Although different cutoff points are used to define virologic failure according to the national guidelines, one study done at Jimma, Southwest Ethiopia, reported a 5.3% virologic failure at 6 month of treatment (defined as viral load >1000 copies per milliliter of plasma) [23]. Meanwhile, a study in Nigeria, Kenya and Cameroon have found a 23.4%, 24.6% and 23.2% virologic failure respectively (defined as viral load > 400 copies per mililiter of plasma). In the study done in Nigeria, poor immune-virologic outcomes were associated with younger age at ART initiation, male sex, hemoglobin, poor adherence measured by pharmacy refill record and low educational status. Treatment failure is found to be significantly associated with young age, unsatisfactory adherence, & anemia in Kenya and Cameroon [29, 30, 31].

Several studies have identified non-adherence, low baseline CD_4_ cell count, rate of CD_4_ decline, prior exposure to ARVs and treatment interruptions as determinants of treatment failure [32, 33, 34]. Of these, non-adherence has been shown to be the major determinant of antiretroviral treatment despite the use of different measure of adherence. The correlation between HIV providers' assessments of patient adherence, documented in the medical record, and treatment failure is unknown. Moreover, others have questioned the ability of HIV clinicians to estimate patients’ adherence accurately [35]. Prior exposure to ARVs, prior duration of prophylaxis or treatment and treatment interruptions lead to monotherapy, particularly, with drugs of long half-life. [32, 33]

Baseline CD_4_ cell count is another important determinant factor for antiretroviral treatment failure. It is known to increase the risk and episodes of opportunistic infections and high attrition rate [26]. In addition to the factors mentioned earlier, a systematic review by Desta identified older age, occurrence of opportunistic infections, the presence of clinical symptoms, co-infections with Hepatitis C virus and human T-cell lymphotropic virus (HTLV) type 1 and 2 as factors associated with treatment failure [20]. A case control study among Tuberculosis (TB) patients in Addis Ababa found that advanced HIV infection (WHO stage 3 & 4), baseline body mass index (BMI) <18.5 kg/m^2^ and baseline CD_4_ cell count were found to be associated with treatment failure in this study [36]. The occurrence of latent and clinical TB, while on antiretroviral treatment, was not found to be associated with treatment failure in studies done in South Africa and Addis Ababa [32, 36]. But, studies from Uganda, Cameroon and India revealed that TB co-morbidity significantly influences virologic failure [31, 37, 38].

A cross sectional studies from Kenya and Cameroon found that duration of antiretroviral treatment was not associated with treatment failure, while a retrospective cohort analysis from Uganda reported that the level of treatment failure was twice at 24 months compared to 12 months of treatment among cohorts [30, 31, 38]. In another study from India, being on ART for more than 3 years was associated with immunological failure [39].Under nutrition, defined as low baseline BMI < 18kg/m^2^ or change in body weight from the baseline, and advanced WHO clinical stage are also additional predictors of treatment failure. These factors are related to each other and their combination weakens the host immunity by predisposing it to different opportunistic infections [28, 36, 37]. The type of initial ARV drug used (usually, NNRTIs and Zidovudine (AZT)) are also other predictive factors [28, 32, 34]. Among the demographic factors, the association of age with treatment failure is controversial as different studies found different results [20, 29, 30, 31, 40]. A case control study from Burkina Faso showed that men were found to be at increased risk of developing treatment failure after adjustment for age, level of education, baseline CD_4_ cell count, first and current antiretroviral regimens and time on ART [41]. Supporting this finding, men on ART appeared to be more vulnerable to virologic failure than women from a retrospective cohort study in Nigeria [29].

Given the limited laboratory facilities, the diagnosis of first-line antiretroviral treatment failure and the initiation of second-line treatment are largely dependent on clinical and immunologic assessments which are less sensitive and predictive of virologic failure. This study aimed to identify factors that predict Antiretroviral Therapy (ART) failure, which will provide information for clinicians for appropriate decision and management of patients on antiretroviral treatment. In addition, this particular study highlighted important factors apart from the already known factors investigated in other study settings. Program planners and clinicians will also use the findings to develop diagnostic algorithm to prioritize patients who need targeted viral load monitoring, considering the available resources.

**Fig 1:**
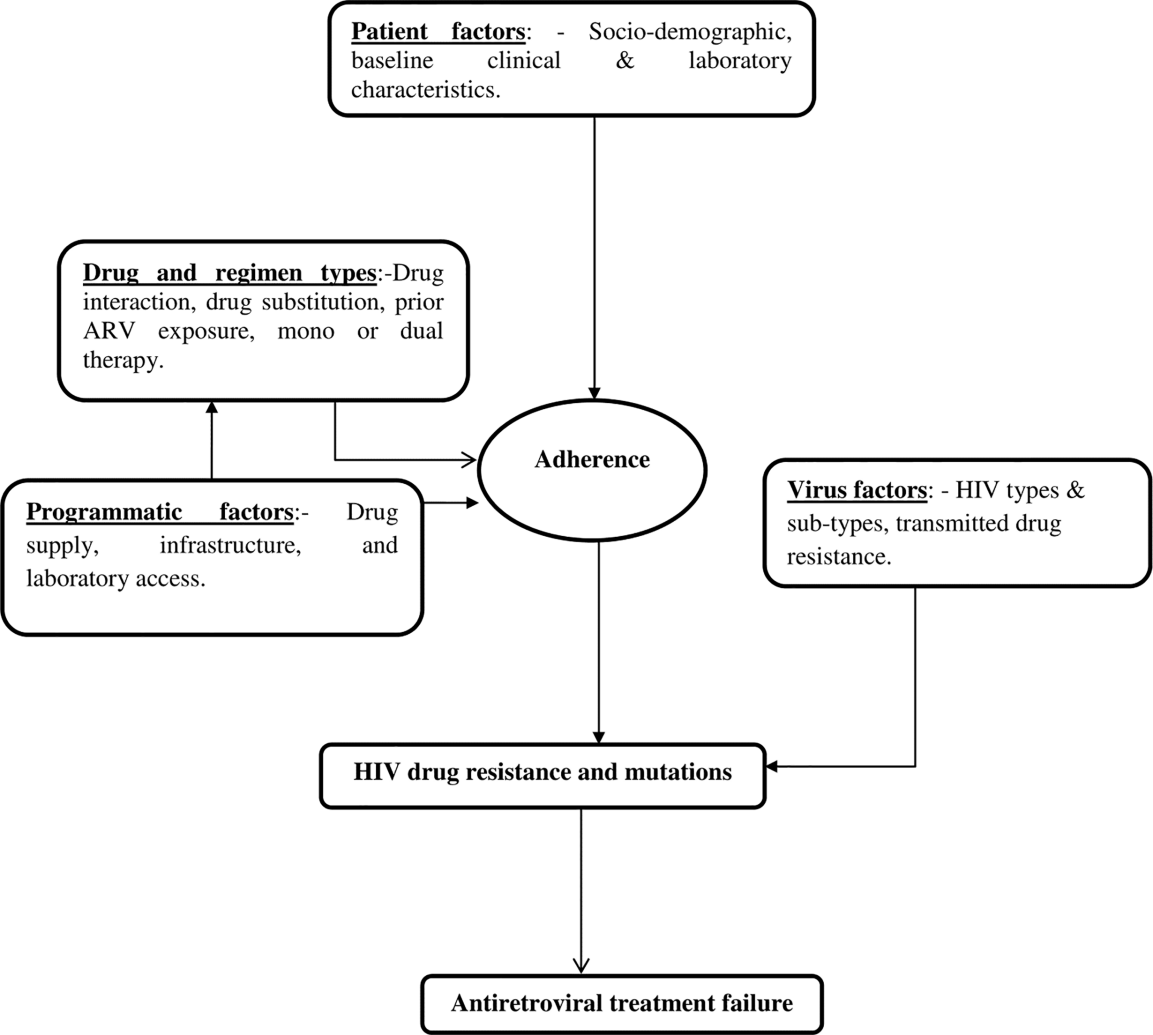
Conceptual framework of risk factors and predictors of antiretroviral treatment in HIV-infected clients, Woldia—Ethiopia, 2014.

The factors include patients related factors, virus related factors, programmatic and drug factors leading directly and indirectly to antiretroviral treatment failure (Fig 1). The objective of this study is to determine factors that predict first-line antiretroviral therapy failure among HIV-Infected Adult clients at Woldia Hospital, Northeast Ethiopia.

## Methods and materials

### Study design

Facility based case-control study of HIV-infected adults on ART and on follow up was carried out at Woldia Hospital from November to December 2014. The study got ethical approval from Wollo University, College of Medicine and Health Sciences. Cases were adult patients who experienced first line regimen failure and switched to second-line regimen and on active follow up while controls were those adult patients on a non-failing first-line regimen for 36 months and above and on active follow up. Cases were required to have been on ART for at least 6 months before switching to second-line regimen.

### Study area and period

The study was conducted from November1 to December 30/ 2014 at Woldia hospital in Woldia town, 521 kilometers northeast of the capital Addis Ababa and having an estimated population of 67,760 [42]. Woldia hospital is one of the public hospitals providing comprehensive service for a catchment population of 1.5 million with 116 beds. The ART clinic services started in 2005 and serve a diverse patient population from neighboring regions (Afar &Tigray). While this study was conducted, the clinic was following the 2008 national ART guideline for treatment of HIV infected patients. As per the guideline, patients were put on first-line ART consisting of two NRTIs and one NNRTI with a monthly follow up for the first six months and every 2–3 months thereafter. CD_4_ cell count was measured every six months and assessment of opportunistic infections and side effects done every visit. Second line regimen consists of boosted protease Inhibitor after a failing first-line regimen. Viral load testing is available at the Bahirdar regional health research laboratory, 360 kilometers Northwest of Woldia, for a limited number of patients suspected of treatment failure. The hospital uses clinical assessment and immunologic evaluation to identify ART failure.

According to the monthly report of the hospital, as of October 2014, 9076 HIV infected peoples were ever enrolled, of whom 6272 started antiretroviral treatment. There were 3713 clients, currently, on ART, of whom 3599 were on first line and 114 were on second line regimen [43].

### Source and study population

HIV-infected adult patients, who were put on second-line and first-line ART based on the national guideline, were the source population for the cases and controls, respectively. The study population, for cases, was sampled from the list of patients on active follow up and taking second-line regimen. Controls were sampled from patients taking a first-line regimen for 36 months and above and on active follow up. Data relevant to this particular study was collected from eligible participants and their medical record simultaneously.

Clients initiated for first-line ART at Woldia Hospital were included on the study, where as those with serious illness and with incomplete relevant data from medical record were excluded.

### Sample size determination

Sample size was calculated for the known significantly associated independent variables: non-adherence, CD_4_ cell count ≤ 100, treatment interruption and prior exposure to ARV drug. Then, the largest sample size in most literatures was found to beCD_4_ cell count ≤ 100, as independent variable was used. The sample size was calculated using Epi Info version 7 considering the following parameters: proportion of CD_4_ cell count ≤ 100 (58.9% in cases and 37.1% in controls) [16], 5% margin of error, 80% power, a case to control ratio of 1:4 and by using a two population proportion formula. The calculated sample was 279 (56 cases and 223controls). By adding a 10% non-response rate, the resulting minimum sample size was 307 (62 cases & 245 controls).

### Sampling procedures

**Fig 2:**
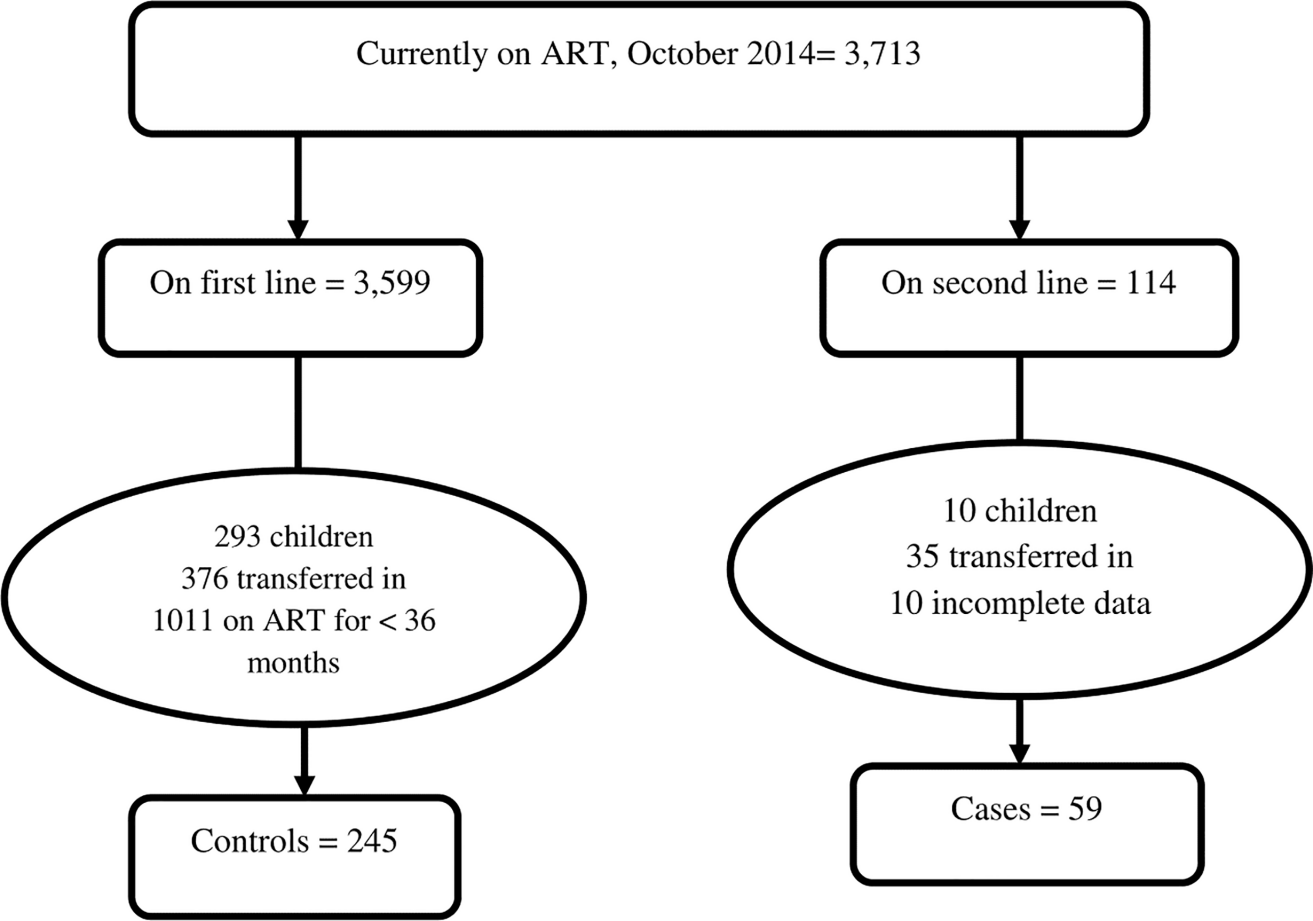
Enrollment of HIV infected adults in the study, Woldia Hospital, 2014.

There were 114 adults currently on second line ART by the end of October 2014. Of these, 35 were transferred in from other health facilities. All the remaining 69 patients who fulfilled the inclusion criteria were included in the study without sampling for their relative small number. Out of whom, 10 were excluded because of the incompleteness of relevant clinical data form their medical record. By the end of October 2014, 3,306 adults were currently on first line ART. The average daily patient flow for drug refill was 55, during the month preceding the data collection. During the data collection period, there were 38 working days and making a total of 2,090 study population from which the sample was taken. The required number of controls for this study (n = 245) was sampled by systematic random sampling from the 2,090 patients who came for refill during the data collection period (Fig 2). At the time of entry to the study selected controls were assessed by clinical and immunological parameters to make sure that they are on a non-failing first-line regimen. When found to have or suspected of treatment failure, the next patient was enrolled to the study. Patients both on first-line and second-line regimen came for medication refill every month or maximum every two months. Thus the study subjects had at least one scheduled clinic appointment during the study period.

### Study variables

The dependent variable for this study is occurrence of antiretroviral treatment failure. For this study, antiretroviral therapy treatment failure was defined as the fulfillment of clinical and/or immunological criteria set by WHO. This was determined by reviewing whether the client was diagnosed with treatment failure or not on his/her medical record. The independent variables include: Socio-demographic variables (age at enrollment, sex), HIV sero-discordance and disclosure, body weight change, baseline CD_4_ cell count, baseline body mass index, baseline hemoglobin, WHO stage, adherence to ART (of the last 3 visits prior to second-line initiation for cases or prior to the current visit for controls, as determined and recorded by the care providers), functional status, occurrence of TB while on ART, initial ARV regimen, prior ARV exposure, treatment interruption (for 7 and more days and as reported by cases and controls), history of drug substitution, recurrent pneumonia and missed appointment.

### Data collection procedures

The data collection tool was developed from prior studies and adapted to this particular study. A structured interviewer administered questionnaire and patient medical record review was used to collect data on the dependent and independent variables. The Amharic version of the questionnaire was used to collect data from study participants and re-translated back to English version for data entry. Major components of the data collection tool were socio-demographic variables, baseline & follow up clinical and laboratory variables. The data collection tool was pre-tested on 25 participants and, based on the findings, corrections were made. The interview was conducted on the day of a scheduled clinic visit upon getting verbal consent to enroll in to the study. A unique identification number was assigned to sampled cases and controls.

### Data analysis

Data was entered using Epi Info version 7 and was exported to SPSS version 20 for analysis. Data was cleaned and edited by running simple frequencies and cross tabulation before analysis. Mean (Standard Deviation, SD), median (Interquartile Range, IQR) and percentages were used to summarize participants characteristics in each group. A chi-square/Fischer’s exact test and independent t-test were used to compare the differences among the categorical and continuous variables between the two groups. Bivariate and multivariable logistic regression modelswereused to identify predictors of ART failure. Independent variables with p < 0.2 in the bivariate analysis were included in the multivariable analysis. Covariates that were a priori known to be significantly associated with the outcome were included into the model regardless of the level of significance in the bivariate analysis. Covariates in the final model were selected by backward step-wise selection procedure. Model of goodness of fit was done by Hosmer-Lemeshow goodness of fit test. Crude and adjusted Odds Ratio with 95% confidence intervals was computed and statistical significance was considered with two sided P-value < 0.05.

### Data quality management

Quality of data was assessed before, during and after data entry. Every filled data collection tool was thoroughly checked for completeness and consistency at the data collection point by the supervisor and before entry by the principal investigator.

## Results

### Socio-demographic characteristics of the study participants

A total of 304 participants (245 controls and 59 cases) were included in this study. The response rate for controls was 100% and 95% for cases. The median age for cases was 33 years (IQR: 27–38) and for controls was 32 years (IQR: 28–40). There were more female than male clients in the control group accounting two third, while the proportion of female and male is approximately equal among cases. Eighty percent of the cases had some educational background (elementary and above) while half of the controls didn’t write or read. Fifty six percent of the cases were unemployed compared to one third among controls [Table 1].

**Table 1:** Comparison of socio-demographic characteristics of cases and controls, Woldia Hospital, 2014.

Variables	Cases n (%)	Controls n (%)
Age (median)	33 (27–38)*	32 (28–40)*
• 18–24	6 (10.2)	18 (7.3)
• 25–34	26 (44.1)	115 (46.9)
• 35–44	23 (39.0)	75 (30.6)
• 45+	2 (6.8)	29 (15.1)
Sex		
• Female	30 (50.8)	163 (66.5)
• Male	29 (49.2)	82 (33.5)
Marital status		
• Single	5 (8.5)	14 (5.7)
• Married	30 (50.8)	122 (49.8)
• Divorced/separated	16 (27.1)	69 (28.2)
• Widowed	8 (13.6)	40 (16.3)
Educational level		
• Don’t write or read	12 (20.3)	110 (44.9)
• Elementary	23 (39.0)	95 (38.8)
• Secondary	15 (25.4)	24 (9.8)
• College diploma & above	9 (15.3)	16 (6.5)
Employment status		
• Farmer	8 (13.6)	71 (29.0)
• Unemployed	33 (55.9)	74 (30.2)
• Employed	18 (30.5)	100 (40.8)
Monthly income (mean)	1464 ± 1401	894 ± 909
Religion		
• Orthodox Christian	44 (74.6)	191 (78.0)
• Muslim	13 (22.0)	50 (20.4)
• Others	2 (3.4)	4 (1.6)
Disclosure to partner		
• Yes	35 (59.3)	142 (58.4)
• No	3 (5.1)	8 (3.3)
• Not applicable	21 (35.6)	95 (38.4)
		

*Inter-quartile range

### Clinical characteristics of the study participants

Voluntary counseling and testing (VCT) was the most common point of entry for HIV testing. HIV sero-status disclosure and discordance rate was similar among the two cohorts. ARV treatment interruption was higher among cases than controls (16.4% vs. 1.2%). There was no difference in the history of prior ARV exposure before starting ART among cases and controls. The median peak CD_4_ count for controls was higher for controls and the mean time required attaining the peak CD_4_ count was also longer for controls compared to cases. Additionally, the mean duration of months on first line ART was shorter for cases as compared to controls.

There was a difference in baseline Body Mass Index (BMI) between the two cohorts. Among cases 37 (62.7%) had a baseline BMI < 18.5, whereas 109 (44.5%) of controls had a baseline BMI < 18.5. A higher proportion of cases (27.1%) had negative or no change in weight from baseline in the first 12 months of ART compared to controls (15.1%). Regarding the initial ARV regimen type, the commonest first line regimens were Stavudine/Lamivudine/Nevirapine, 40%; Zidovudine/Lamivudine/Nevirapine, 27.7%; Stavudine/Lamivudine/Efavirenz,10.5% followed by Zidovudine/Lamivudine/Nevirapine, 7.2%. But there was no significant difference in the type of NRTIs (Stavudine vs. Zidovudine) and NNRTIs (Nevirapine vs. Efavirenz) used in the first line regimen between the cases and controls.

Compared to controls a higher proportion of cases had history of incident tuberculosis after ART initiation (6.5% vs. 16.5%). In addition cases had slightly higher non-adherence and recurrent pneumonia (more than 2 episodes). Baseline WHO stage, functional status, CD_4_ count, hemoglobin and history of drug substitution were not statistically different among the two cohorts (Table 2).

**Table 2:** Comparison of clinical characteristics of cases and controls, Woldia Hospital, 2014.

Variable	Cases n (%)	Controls n (%)
HIV testing		
· VCT	58 (98.3)	226 (93.4)
· PITC/check up	1 (1.7)	19 (6.6)
Discordance with partner		
· Yes	25 (42.4)	95 (38.8)
· No	10 (16.9)	41 (16.7)
· Unknown/Not applicable	24 (40.7)	109 (46.4)
Prior ARV exposure		
· Yes	0	1 (0.4)
· No	59 (100)	244 (99.4)
ART interruption		
· Yes	10 (16.9)	3 (1.2)
· No	49 (83.1)	242 (98.8)
Baseline WHO stage		
· Early (stage 1 &2)	16 (27.1)	76 (31.0)
· Advanced (stage 3 & 4)	43 (72.9)	169 (68.9)
Baseline functional status		
· Working	29 (49.2)	141 (57.6)
· Ambulatory	27 (45.8)	90 (36.7)
· Bedridden	3 (5.1)	14 (5.7)
Baseline CD_4_ count (median)	105 (60–174)*	131 (72.5–189.0)*
· < 100	28 (47.5)	91 (37.1)
· ≥ 100	31 (52.5)	154 (62.9%)
Peak CD4 count (median)	230 (23–387)*	463 (348.5–577)
Mean time to peak CD_4_ count	16.5 ± 10.8	37.2 ± 19.0
Baseline hemoglobin (median)	12.7 (11.4–13.7)*	12.9 (11.5–14.0)*
· < 10	4 (6.8)	25 (10.2)
· ≥ 10	55 (93.2)	220 (89.8)
Baseline BMI (median)	17.9 (17.0–19.5)*	18.7 (16.9–20.2)*
· < 18.5	37 (62.7)	109 (44.5)
· ≥ 18.5	22 (37.3)	136 (55.5)
Weight change		
· Negative/No change	16 (27.1)	37 (15.1)
· Positive	43 (72.9)	208 (84.9)
Initial ARV regimen		
Stavudine vs. Zidovudine		
· Stavudine based	31 (52.5)	139 (56.7)
· Zidovudine based	16 (27.1)	89 (36.3)
Nevirapine vs. Efavirenz		
· Nevirapine based	32 (54.2)	183 (80.3)
· Efavirenz based	15 (25.4)	45 (18.4)
Drug substitution		
· One or more	43 (72.9)	157 (64.1)
· None	16 (27.1)	88 (35.9)
Adherence status		
· Good	55 (93.2)	245 (100)
· Fair/Poor	4 (6.8)	0
Recurrent pneumonia		
· Yes	4 (6.8)	1 (0.4)
· No	55 (93.2)	244 (99.6)
Incident Tuberculosis		
· Yes	10 (16.9)	16 (6.5)
· No	49 (83.1)	229 (93.5)
Mean time on 1^st^ line ART	48.4 ± 20.7	76.5 ± 19.1

* Inter-quartile range.

BMI: Body Mass Index; VCT: Voluntary Counseling & Testing; ARV: Antiretroviral; ART: Antiretroviral Therapy; PITC: Provider-Initiated Testing & Counseling

### Predictors of treatment failure among HIV infected adults

The bivariate analysis showed that age at ART enrollment was not associated with ART failure. Females were less likely to develop treatment failure compared to males. HIV infected adults who had some educational level above elementary had increased risk of developing ART failure. The risk appeared to be higher for those with tertiary level education. Unemployed patients had 2.5 times increased risk of ART failure (COR = 2.48; 95% CI 1.296, 4.737).

Baseline WHO stage, CD_4_ count, hemoglobin and history of drug substitution did not show any significant association with the occurrence of ART failure. Compared to the reference group, patients with baseline BMI < 18.5 kg/m^2^ and with negative or no change in weight from the baseline within 12 months of ART initiation had twice the risk of developing ART failure. Patients with incident tuberculosis after initiation of ART had three times increased risk of developing treatment failure.

Patients with high peak CD_4_ count and with longer time to attain peak CD_4_ count had decreased risk of treatment failure. The median peak CD_4_ count and mean duration to achieve peak CD_4_ count was 230 cells/μl and 16.5 months for cases and 463 cells/μl and 37.2 months for controls. In the bivariate analysis the type of initial ARV regimen used (d4T vs. AZT or NVP vs. EFV) did not show any significant association with treatment failure. The risk of treatment failure was lower among patients with longer duration on first line ART (COR = 0.93; 95% CI 0.915. 0.951).

ARV treatment interruption and occurrence of recurrent pneumonia were also significantly associated with treatment failure but the precision of estimation was weak as evidenced by the wide confidence interval [Table 3].

**Table 3:** Predictors of antiretroviral treatment failure, Woldia hospital, 2014.

Variables	Cases	Controls n (%)	COR (95% CI)	p-value	AOR (95% CI)	p-value
	n (%)					
Median age at enrollment	33	32	0.98 (0.950, 1.017)	0.315		
• < 35	38 (64)	151 (62)	1.13 (0.623, 2.036)	0.693	0.834(0.270,2,579)	0.753
• ≥ 35	21 (36)	94 (38)	1		1	
Sex						
• Female	30(51)	163(66)	0.52 (0.293, 0.923)	0.026	0.486(0.154,0.538)	0.219
• Male	29(49)	82 (34)	1		1	
Educational level						
• Don’t write or read	12 (20)	110 (45)	1		1	
• Formal education	38 (65)	119 (49)	2.93(1.455,5.888)	0.001	5.15(1.534,17.276)	0.008
• Tertiary	9 (15)	16 (6)	5.16(1.876,14.710)	0.003	6.60(0.921,47.324)	0.06
Employment status						
• Farmer	8 (14)	71 (29)	0.63 (0.258, 1.519)	0.3	0.996(0.231,4.298)	0.996
• Unemployed	33(56)	74 (30)	2.48 (1.296, 4.737)	0.006	4.93(1.493,16.305)	0.009
• Employed	18(30)	100(41)	1			
Drug substitution						
• Yes	43 (73)	157 (64)	0.66 (0.355, 1.247)	0.203	*	
• No	16 (27)	88 (34)	1			
					
Treatment interruption						
• Yes	10 (17)	3 (1)	16.46(4.370, 62.012)	<0.001	11.75(1.38,99.904)	0.024
• No	49 (83)	242 (99)	1		1	
Baseline WHO stage						
• Advanced stage	43 (73)	169 (69)	1.20 (0.641, 2.280)	0.558	0.815(0.255,2.605)	0.73
• Early stage	16 (27)	76 (31)	1		1	
Baseline CD_4_ count (median)	105 (IQR = 60–174)	131 (IQR = 72.5–189.0)91 (37)	0.99 (0.994, 1.002)	0.3		
	28 (47)	154 (63)	1.53 (0.862, 2.711)	0.147	0.653(0.223,1.915)	0.438
• < 100	31 (53)		1		1	
• ≥ 100						
Peak CD_4_ count (median)	230 (IQR = 123–387)	463 (IQR = 348.5–577)	0.992 (0.990, 0.995)	<0.001	0.993(0.990,0.996)	<0.001
Time to peak CD_4_ count (mean)	16±11	37±19	0.89 (0.867, 0.927)	<0.001	*	
Baseline Hemoglobin (median)	12.7 (IQR = 11.4–13.7)	12.9 (IQR = 11.5–14.0)	1.06 (0.961, 1.181)	0.228	*	
	4 (7)	25 (10)				
• < 10	55 (93)	220 (90)	0.64 (0.214, 1.915)	0.425		
• ≥ 10			1			
Baseline BMI (Median)	17.9 (IQR = 17.0–19.5)	18.7 (IQR = 16.9–20.2)				
	37 (63)	109 (45)		0.019	2.75(1.012,7.457)	0.047
• < 18.5	22 (37)	136 (55)	2.10 (1.169, 3.766)			
• ≥ 18.5			1			
Weight change						
• Negative/No change	16 (27)	37 (15)	2.09 (1.068,4.096)	0.031	1.242(0.322,4.791)	0.753
• Positive						
	43 (73)	208 (85)	1		1	
Initial ARV regimen						
Stavudine Vs. Zidovudine						0.028
• Stavudine based	31 (53)	139 (57)	1.24 (0.642,2.399)	0.522	3.47(1.143,10.555)	
• Zidovudine based	16 (27)	89 (36)	1		1	
Nevirapine Vs. Efavirenz						0.817
• Nevirapine based	32 (54)	183 (75)	0.53(0.261,1.050)	0.069	0.865(0.253,2.962)	
• Efavirenz based	15 (25)	45 (18)	1		1	
Incident TB						
• Yes	10 (17)	16 (7)	2.92 (1.25, 6.82)	0.013	1.494(0.299,7.464)	0.624
• No	49 (83)	229 (93)	1		1	
Recurrent pneumonia						
• Yes	4 (7)	1 (0.4)	17.75(1.945,61.884)	0.011	23.02(1.644,322.47)	0.02
• No	55 (93)	244(99.6)	1		1	
Duration on first line ART	48±21	76±19	0.933(0.915,0.951)	<0.001	0.923(0.893,0.954)	<0.001

* Variables not included in the multivariate analysis; AOR: Adjusted Odds Ratio; COR: Crude Odds Ratio.

In the multivariate analysis initial ARV regimen type, unemployment, having formal education (Grade 1–12), low baseline BMI, peak CD_4_ count and duration on first line ART were independent predictors of ARV treatment failure after controlling for other factors. Receiving stavudine based first line regimen had three fold increased risk of treatment failure compared to Zidovudine based regimen. In addition peak CD_4_ count achieved during the first line ART and low baseline BMI (< 18.5) were also significant predictors of treatment failure. Patients who attain high peak CD_4_ count were less likely to develop treatment failure (AOR = 0.993; 95% CI 0.990, 0.996). Although the time required to attain peak CD_4_ count was associated with treatment failure in the bivariate analysis, this effect couldn’t be demonstrated in the multivariate model.

Patients who were unemployed and with formal education had increased risk of ARV treatment failure. Furthermore duration on first line ART was another factor significantly associated with treatment failure. Patients who failed on ART had shorter duration on first line therapy. The mean duration on first line ART was 48.4 months for cases and 76.5 months for controls (AOR = 0.923; 95% CI 0.893, 0.954). History of treatment interruption and the occurrence of recurrent pneumonia were also associated with treatment failure but the power of estimation was weak as seen with the very wide confidence interval.

Variable that were not significantly associated with treatment failure in the multivariable analysis includes age, sex, weight change, incident TB, baseline CD_4_ count and WHO clinical stage [Table 3].

## Discussion

Females account to two third of the adults on treatment and majority of the index patients has disclosed their HIV status to their partners. Similar finding was reported from other study in Ethiopia [16]. Different studies have found that men were more likely to develop treatment failure [28, 29, 41] as most men are diagnosed and present to health care lately [15]. But in this study the association of sex with treatment failure was not statistically significant.

The median age of the study participants was comparable to a finding from Debremarkos hospital study and close to half of the participants were in the age group 25–34 years [16]. Various studies have revealed that younger age groups (usually < 35 years) were at increased risk of treatment failure [29, 30, 31, 40]. In contrast a systematic review by Misgena found that old age was associated with treatment failure [20]. However, the association of age with treatment failure could not be demonstrated in this study.

Results from this study revealed that duration of first line ART regimen was an important determinant of treatment failure. Patients, who were on their first line regimen for longer period, appear to have low risk of treatment failure. A large observational cohort study by Dragsted et al have found that a gradual and significant decline in the rate of immunologic failure over time. In fact the trend over time showed a 28% decrease with each additional year since the initial immunologic response [27]. Contrary to this, a cross sectional study from India by Prabhakar et al found that duration of more than 3 years on ART was significantly associated with immunological failure [39].

Stavudine use as NRTI backbone was significantly associated with a threefold increased risk of treatment failure compared to Zidovudine based regimen. However, a study from Kenya showed that Zidovudine based regimen was associated with treatment failure partly explained by the early adverse effects which includes nausea and vomiting that potentially affect treatment adherence [34]. Ethiopia has already phased out stavudine based regimen from the HIV treatment program based on WHO recommendation due to the long term toxicity but the increased risk of treatment failure associated with stavudine based regimen can also be a reason for the phase out. The choice of NNRTIs (NVP vs. EFV) was not significantly associated with treatment failure in this particular study. This finding is similar to a study from Kenya [34]. While a study from South Africa revealed a 2.5 times increased risk of treatment failure in the NVP based group compared to EFV based group [32]. Another study from India showed that failure was significantly higher in the EFV based regimen cohort [28].

Various studies have shown that low baseline CD4 count and advance disease stage were significant predictors of treatment failure explained by the frequent occurrence of opportunistic infections that leads to disease progression subsequently increased risk of failure [25, 27, 32, 39]. More than two third of the patients in this cohort had advanced disease stage at enrollment and their CD_4_ count at ART initiation was low (mean CD_4_ = 132 cells/microliter). Interestingly, however, both advanced WHO clinical stage and low CD_4_ count were not significantly associated with treatment failure. This is similar to findings from Kenya where both WHO stage [30, 34] and baseline CD4 count were not associated with treatment failure [34]. Rather peak CD_4_ count was found to be the strongest predictor of treatment failure. Low peak CD_4_ count was significantly associated with increased risk of failure. Singh et al in their study found that the mean peak CD_4_ count for the non-failure group was twice that of the failure group [37]. This finding could be explained by the fact that the rate of immunologic recovery in the first year of ART among patients with low baseline CD_4_ count was equal or greater compared to those with high baseline CD_4_ count. However, their CD_4_ count remains low (< 200 cells/microliter) for longer period increasing the risk of morbidity and subsequent disease progression [25].

Unemployed patients had about 5 times higher risk of treatment failure compared to employed patients. The possible explanation could be those patients who were unemployed may have low income that hinders them from getting the opportunity for early and better care and support. This is in agreement with a finding by Melsew et al [16]. The other interesting finding from this study was the association between educational level and treatment failure. Compared to the illiterate and those with tertiary level and formal education between 1–12 grades had increased odds of treatment failure. A study in Southern Ethiopia by Teshome and Assefa found that those with a higher education level were at increased risk of treatment failure [44]. A possible explanation could be the association of educational level with employment status. In this study more than half of the unemployed patients had primary and secondary educational level. A second explanation could be the association between adherence to treatment and educational level. However, inconclusive findings were reported regarding this association from a systematic review by Peltzer et al [45]. A third explanation could be the level of counseling offered to educated patients assuming that they know better & the associated stigma and discrimination.

Of the well-known factors, adherence was one of the strongest predictor of treatment failure [33, 34, 40]. In this study there was a higher non-adherence in the failure group as evidenced by the Fischer’s exact test but the effect of non-adherence on the treatment failure could not be explored in the final regression model because of the very low number of participants reported to have non-adherence. In addition the method of adherence measurement used was provider’s assessment and record of adherence level at each clinic visit and the correlate of such method of adherence with treatment failure is unknown [35].

Prior ARV exposure for PMTCT purpose was reported to be very low in this cohort probably reflecting the low coverage and enrollment to PMTCT service in the early period of the HIV treatment program in Ethiopia. Studies from other African countries have found that an increased risk of treatment failure among patients with prior ARV exposure [32, 33]. In addition ARV treatment interruption for 7 or more days was significantly associated with treatment failure.

The occurrence of recurrent pneumonia (> 2 episodes) was another predictor of treatment failure. Similar finding was reported from other study [16]. The occurrence of incident tuberculosis (TB) was not a significant predictor of treatment failure. Tuberculosis infection is one of the commonest opportunistic infections among HIV infected adults regardless of immunologic status. The occurrence of tuberculosis during ART has multiple effects including pill burden, drug-drug interaction and overlapping toxicities which influence adherence [5, 7]. A study in Addis Ababa has found that TB didn’t influence ART outcome [36]. In agreement with this TB was not associated with treatment failure [32]. In contrast other studies have showed that patients with incident TB had increased risk of treatment failure [37, 46].

Some studies have revealed that patients with low baseline BMI (< 18.5) and negative or no change in weight have weakened immunity and blunted immune response and increased risk of treatment failure [16, 28, 36, 37]. However, in this study low baseline BMI not weight change which was found to be associated with the occurrence of treatment failure. This may indicate that the level of nutritional status determines the immune response than the trend in weight change.

Regardless to the study’s strengths and the inevitable intrinsic limitations of using case control study for the study, the following limitations worth mentioning. One of the limitations of this study was the determination of treatment failure through clinical and/or immunological criteria due to the absence of viral load testing to confirm its presence. In addition, the use of adherence measurement based on health care provider’s assessment inadequately detects poor adherence even though this method used to be the usual clinical practice in the Ethiopian context. Moreover, the exclusion of patients who were initiated for ART in other health facilities may introduce bias. Not standardizing the cases and controls in terms of duration of treatment may introduce bias in predicting the outcome.

## Conclusion and recommendations

This study yielded a valuable insight to the various predictors of treatment failure. Apart from the well-known factors identified elsewhere, this study have found a novel risk factor of peak CD_4_ count achieved during the first line therapy that has not been demonstrated in the Ethiopian context. In addition it was revealed that the risk of treatment failure in patients with an initial response to ART decreases with a longer duration on first line treatment.

Another interesting finding was the negative association between educational level and immunologic response. Being unemployed, stavudine based initial regimen, having recurrent pneumonia and history of antiretroviral treatment interruption for more than 7 days were also significantly associated with increased risk of treatment failure. While low baseline CD_4_ count, advanced WHO stage and occurrence of incident TB were not significant predictors of treatment failure in this study.

With a maturing HIV treatment program in Ethiopia monitoring of patients on first line treatment to identify those who are more likely to develop treatment failure is highly crucial. Clinicians should exert their effort to maintain patients on their initial regimen for as long as possible duration with appropriate care and close follow-up. Among the different markers of treatment failure the level of peak CD_4_ cell count achieved during ART should be monitored closely. In addition, programs managers should have their own efforts in improving socio-economic status of HIV infected patients through different livelihood initiatives should be strengthened. Moreover, besides the clinical care provided to patients on treatment, the integrated nutritional counseling and supplementary food support program with in the HIV treatment platform should be further strengthened. To enhance the evidence in this regard, some of the predictive factors identified in this study needs to be further explored with longitudinal studies and larger sample size to ascertain their association, by researchers. In addition, as cumulative evidences revealed from various studies adherence is the most important determinant of treatment failure, a validated measure of adherence shall be considered in future studies.

## Supporting information

S1 File Data collection tools. (PDF)

## References

[1] Joint United Nations Programme on HIV/AIDS (UNAIDS). Global AIDS Update: unaids.org. 2016. 12349391

[2] Federal HAPCO. Country progress report on HIV/AIDS response, 2014.Addis Ababa, Ethiopia.

[3] CSA. Ethiopia Demographic and Health Survey 2011. March 2012.Addis Ababa, Ethiopia.

[4] Palmisano L, Vella S. A brief history of antiretroviral therapy of HIV infection: success and challenges. *HIV Therapy Today*. 2011; 47(1):44–48. 10.4415/ANN_11_01_10 21430338

[5] WHO. Consolidated Guidelines on the Use of Antiretroviral Drugs for Treating and Preventing HIV Infection, recommendations for Public Health Approach. June 2013. 24716260

[6] WHO. Global Update on HIV Treatment 2013: Results, Impacts and Challenges. June 2013.

[7] FMOH. Guidelines for the management of opportunistic infections and antiretroviral treatment in adolescents and adults in Ethiopia. March 2008.

[8] Amhara HIV/AIDS Prevention and Control Coordination Office. Accessed on July 19, 2014). Available from: www.etharc.org/Amhara/Resource/HIV Facts and Figures.

[9] EHNRI. HIV Drug Resistance (HIVDR) Early Warning Indicators EWIs) Survey in Ethiopia. October 2013/14. Addis Ababa, Ethiopia.

[10] Harries AD, Zachariha R, van Oosterhout JJ, Reid SD, Hosseinipour MC, Arendt V, et al Diagnosis and management of antiretroviral therapy failure in resource-limited settings in sub-Saharan Africa: challenges and perspectives. Lancet Infect Dis. 2010; 10:60–65. doi: 10.1016/S1473-3099(09)70321-4 20129150 10.1016/S1473-3099(09)70321-4

[11] WHO. WHO case definitions of HIV for surveillance and revised clinical staging and immunological classification of HIV-related diseases in adults and children. 2007. Geneva, Switzerland.

[12] Gilks C, Pillay D, Kityo C, Munderi P, Hakim J, Kaleebu P, et al Emergence and evolution of drug resistance in the absence of viral load monitoring during a 48 weeks of Combivir/Tenofovir within the DART trial. *AIDS* 2006; 20(10):1391–1399. doi: 10.1097/01.aids.0000233572.59522.4516791013

[13] Cozz-lepri A, Paredes R, Phillips AN, Clotet B, Kjær J, Von Wyl V, et al The rate of accumulation of non-nucleoside reverse transcriptase inhibitor (NNRTI) resistance in patients kept on a virologically failing regimen containing an NNRTI. HIV Medicine. 2012; 13: 62–72. doi: 10.1111/j.1468-1293.2011.00943.x 21848790 10.1111/j.1468-1293.2011.00943.x

[14] Kifle M. FMOH-Viral Load costing presentation. April 2013. (Cited on May 2014) Available from: http://www.aslm.org/resource-centre/hiv-viral-load-testing/viral-load-testing-consultation-meeting-presentations-april-2013/

[15] Assefa Y, Kiflie A, Tesfaye D, Haile Mariam D, Kloos H, Edwin W, et al Outcomes of antiretroviral treatment program in Ethiopia: Retention of patients on care is a major challenge and varies across health facilities. *BMC Health Services Research*. 2011;11: 81 doi: 10.1186/1472-6963-11-81 21501509 10.1186/1472-6963-11-81 PMC3094207

[16] Melsew YA, Terefe MW, Tessema GA, Ayele TA. Rate of Immunological Failure and its Predictors among Patients on Highly Active Antiretroviral Therapy at Debremarkos Hospital, North West Ethiopia: A Retrospective Follow up Study. *J AIDS Clin Res*. 2013; 4:211 doi: 10.4172/2155-6113.1000211

[17] Bennett DE, Bertagnolio S, Sutherland D, Gilks CF.: The World Health Organization’s global strategy for prevention and assessment of HIV drug resistance. *Antiviral Therapy*. 2008; 13 suppl 2:1–13. 18578063

[18] WHO. HIV drug resistance report, 2012. (Cited on May 2014) Available from: http://www.who.int/hiv/pub/drugresistance/report2012/en/

[19] Rawizza HE, Chaplin B, Meloni ST, Eisen G, Rao T, Sankale JL, et al: Immunologic criteria are poor predictors of virologic outcome: implications for HIV treatment monitoring in resource-limited settings. Clin Infect Dis.2011; 53 (12): 1283–1290. doi: 10.1093/cid/cir729 22080121 10.1093/cid/cir729 PMC3246873

[20] Misgena DK. The pattern of immunologic and virologic response to Highly Antiretroviral Active Treatment (HAART): Does success bring further challenges? Ethiop. J. Health Dev. 2011;25(1):61–70.

[21] Ferreyra C, Yun O, Elsenberg N, Alonso A, Khamadi AS, Mwan M, et al: Evaluation of clinical and immunological markers for predicting virological failure in HIV/AIDS treatment cohort in Busia, Kenya. PLoS ONE. 2012; 7(11): e49834 doi: 10.1371/journal.pone.0049834 23185450 10.1371/journal.pone.0049834 PMC3504110

[22] Stafford K, Etienne M, Aina O, Lewin S, Bositis A, Bositis C., et al.: Immunological improvement and viral suppression after the initiation of antiretroviral therapy (ART) in Zambia. 4th IAS Conference on HIV Pathogenesis, Treatment and Prevention: Abstract no. CDB292".

[23] Abdissa A, Yilma D, Fonager J, Audelin AM, Christensen LH, Olsen MF, et al: Drug resistance in HIV patients with virological failure or slow virological response to antiretroviral therapy in Ethiopia. BMC Infectious Diseases. 2014; 14:181 doi: 10.1186/1471-2334-14-181 24708645 10.1186/1471-2334-14-181 PMC4234735

[24] Mulu A, Liebert UG and Maier M.: Virological efficacy and immunological recovery among Ethiopian HIV-infected adults and children. BMC Infectious Diseases. 2014; 14:28 doi: 10.1186/1471-2334-14-28 24422906 10.1186/1471-2334-14-28 PMC3900473

[25] Lawn SD, Myer L, Bekker LG and Wood R. CD4 cell count recovery among HIV-infected patients with very advance immunodeficiency commencing antiretroviral treatment in sub-Saharan Africa. BMC Infectious Diseases. 2006; 6:59 doi: 10.1186/1471-2334-6-59 16551345 10.1186/1471-2334-6-59 PMC1435908

[26] Palladino C, Briz V, Bellón JM, Bártolo I, Carvalho P, Camacho R, et al: Predictors of Attrition and Immunological Failure in HIV-1 Patients on Highly Active Antiretroviral Therapy from Different Healthcare Settings in Mozambique. PLoS One. 2013; 8(12): e82718 doi: 10.1371/journal.pone.0082718 24376569 10.1371/journal.pone.0082718 PMC3869714

[27] Dragsted UB, Mocroft A, Vella S, Viard JP.: Predictors of Immunological Failure after Initial Response to Highly Active Antiretroviral Therapy in HIV-1-Infected Adults: A EuroSIDA Study. J Infect Diseases. 2004; 190(1): 148–155. 15195254 10.1086/420786

[28] Rajasekaran S, Jeyaseelan L, Vijila S, Gomathi C, Raja K. Predictors of failure of first-line antiretroviral therapy in HIV-infected adults: Indian experience. AIDS 2007; 21 Suppl 4:S47–53. 10.1097/01.aids.0000279706.24428.78 17620752

[29] Anude CJ, Eze E, Onyegbutulem HC, Charurat M, Etiebet MA, Ajayi S, et al: Immuno-virologic outcomes and immune-virologic discordance among adults alive and on anti-retroviral therapy at 12 months in Nigeria. BMC Infectious Diseases. 2103; 13:113 doi: 10.1186/1471-2334-13-113 23452915 10.1186/1471-2334-13-113 PMC3599241

[30] Hassan AS, Nabwera HM, Mwaringa SM, Obonyo CA, Sanders EJ, Rinke de Wit TF, et al: HIV-1 virologic failure and acquired drug resistance among first-line antiretroviral experienced adults at a rural HIV clinic in coastal Kenya: a cross-sectional study. AIDS Research and Therapy. 2014; 11:9 doi: 10.1186/1742-6405-11-9 24456757 10.1186/1742-6405-11-9 PMC3922732

[31] Meriki HD, Tufon KA, Afegenwi MH, Nyindem BA, Atanga PN, Anong DN, et al: Immuno-haematologic and virologic responses and predictors of virologic failure in HIV-1 infected adults on first-line antiretroviral therapy in Cameroon. Infectious Diseases of Poverty. 2014; 3:5 doi: 10.1186/2049-9957-3-5 24479873 10.1186/2049-9957-3-5 PMC3922096

[32] Datay MI, Boulle A, Mant D and Yudkin P.: Associations with virologic treatment failure in adults on antiretroviral in South Africa. J Acquir Immune DeficSyndr. 2010; 54 (5):489–495. 10.1097/QAI.0b013e3181d91788 20395870

[33] Greig JE, Cros PA, Mills C, Ugwoeruchukwu W, Etsetowaghan A, Grillo A, et al Predictors of Raised Viral Load during Antiretroviral Therapy in Patients with and without Prior Antiretroviral Use: A Cross-Sectional Study. PLoS ONE. 2013; 8(8): e71407 doi: 10.1371/journal.pone.0071407 23967207 10.1371/journal.pone.0071407 PMC3743819

[34] Kowbah CM, Mwangi AW, Koech JK, Simiyu GN and Siika AM.: Factors Associated with First-Line Antiretroviral Therapy Failure amongst HIV-Infected African Patients: A Case-Control Study. *World Journal of AIDS*. 2012; 2:271–278.

[35] Murri R, Ammassari A, Trotta MP, De Luca A, Melzi S, Minardi C, et al: Patient-reported and physician-estimated adherence to HAART. J Gen Intern Med. 2014;19(11):104–110.doi: 10.1111/j.1525-1497.2004.30248.x 15566439 10.1111/j.1525-1497.2004.30248.x PMC1494787

[36] Kassa D, Gebremichael G, Alemayehu Y, Wolday D, Messele T and van Baarle D.: Virologic and immunologic outcome of HAART in Human Immunodeficiency (HIV)-1 infected patients with and without Tuberculosis and Latent TB infection (LTBI) in Addis Ababa, Ethiopia. AIDS Research and Therapy. 2013; 10:18 doi: 10.1186/1742-6405-10-18 23842109 10.1186/1742-6405-10-18 PMC3718701

[37] Agarwal A, Singh A, Chakravarty J, Sundar S, Rai M,: Predictive Markers of Failure of First Line Anti-Retroviral Treatment in HIV Patients in India. J AIDS Clin Res 2013; 4: 210 doi: 10.4172/2155-6113.1000210

[38] Ahoua L, Guenther G, Pinoges L, Anguzu P, Chaix M, Tiec CL, et al: Risk factors for virological failure and subtherapeutic antiretroviral drug concentrations in HIV-positive adults treated in rural northwestern Uganda. BMC Infectious Diseases. 2009; 9:81 doi: 10.1186/1471-2334-9-81 19493344 10.1186/1471-2334-9-81 PMC2701435

[39] Prabhakar B, Banu A, Pavithra HB, Chandrashekhara P and Sasthri S. Immunological failure despite virological suppression in HIV seropositive individuals on antiretroviral therapy. Indian J Sex Transm Dis. 2011; 32(2): 94–98. doi: 10.4103/0253-7184.85412 22021970 10.4103/2589-0557.85412 PMC3195189

[40] Khienprasit, Chaiwarith R, Sirisanthana T and Supparatpinyo K.: Incidence and risk factors of antiretroviral treatment failure in treatment-naïve HIV-infected patients at Chiang Mai University Hospital, Thailand. AIDS Research and Therapy. 2011; 8:42 doi: 10.1186/1742-6405-8-42 22060823 10.1186/1742-6405-8-42 PMC3238297

[41] Penot P, Héma A, Bado G, Kaboré F, Soré I, Sombié D, et al: The vulnerability of men to virologic failure during antiretroviral therapy in a public routine clinic in Burkina Faso. J Int AIDS Soc. 2014; 17(1): 18646 doi: 10.7448/IAS.17.1.18646 24433983 10.7448/IAS.17.1.18646 PMC3895258

[42] Bureau of Finance and Economic Development, Amhara Regional State. July 2014.

[43] Woldia Hospital. Monthly Facility ART report. October 2014.

[44] Teshome W, Assefa A. Predictors of Immunological Failure of Antiretroviral Therapy among HIV Infected Patients in Ethiopia: A Matched Case-Control Study. PLoS ONE 2014; 9(12):e115125 doi: 10.1371/journal.pone.0115125 25536416 10.1371/journal.pone.0115125 PMC4275231

[45] Peltzer K, Pengpid S. Socioeconomic factors in adherence to HIV therapy in low- and middleincomecountries. J Health PopulNutr 2013; 31: 150–170. 10.3329/jhpn.v31i2.16379 PMC3702336 23930333

[46] Assefa A, Gelaw B, Getnet G, Ytayew G,: The effect of incident tuberculosis on immunological response of HIV patients on highly active anti-retroviraltherapy at the university of Gondar hospital, northwest Ethiopia: aretrospective follow-up study. BMC Infectious Diseases 2014 14:468 doi: 10.1186/1471-2334-14-468 25164855 10.1186/1471-2334-14-468 PMC4158052

