# Supplementary material for: Predictors of first-line antiretroviral therapy failure amongst HIV-infected adult clients at Woldia Hospital, Northeast Ethiopia
Source: PLoS One. 2017 Nov 2;12(11):e0187694. doi: 10.1371/journal.pone.0187694 (PMC5667926; doi:10.1371/journal.pone.0187694)
Supplement: S1 File — (PDF) [file pone.0187694.s001.pdf]

**Data collection tool, English version**

This information sheet is prepared with the aim to provide adequate information to the study participants about the objective, benefits and risks of the study before they agree to participate. The study is a case-control study design on predictors of first-line antiretroviral treatment among HIV-infected adult clients at Dessie Referral Hospital. The investigator is Dr. Yohannes Demissie, MPH candidate from Wollo University, College of Medicine and Health Science. The main aim of the study is to identify factors that predict the occurrence of antiretroviral treatment among HIV-infected adults who are on treatment attending chronic HIV care at this clinic. This study will provide additional that help to improve the ART service provision especially those who need appropriate and timely switch to second-line treatment.

The study will be conducted by collecting data by interviewing volunteered clients and reviewing their medical record by trained ART nurses working in the clinic. You will not have any health risk by participating in this study. The only risk you will have is that the interview will take 15-20 minutes. You will not be provided with any incentives for participating in this study but you will be screened for treatment failure and some mental health conditions as part of the study. All records will be kept confidential. No information that identifies individual clients will be filled on the data collection format. Only aggregated data obtained from this study will be shared to the responsible peoples to improve service provision.

You have the full right to refuse to participate in the study. Refusal to participate will not have any consequences on your treatment or any services you wish to get form the clinic or hospital. However, the information that you provide will help greatly to understand more about antiretroviral treatment failure and the factors associated with it. I appreciate your help in becoming volunteer and respond to the questions. If you are clear with the information provided and agree to participate, we can proceed to the interview.

Thank you.

Are you willing to participant in the study? 1) Yes 2) No

If you have any questions or something not clear feel free to ask. You can contact the investigator. Below is the investigator’s name and address.

Dr. Yohannes Demissie, Management Sciences for Health, Dessie sub-regional office

(Mobile: 0911 80 44 59)

**Part one: Interview questions for interviewing clients who volunteer to participate in the study.**

Medical Record Number------------------------ Date of interview (dd/mm/yyyy) ----------------in EC.

Participant code number -------------------------- (Three digits; start with 001)

**Note:Please circle the response of the participant among the alternatives or otherwise write in the space provided.**

| 1. Socio-demographic characteristics | | | |
|---|---|---|---|
| No. | Question | Response | Code |
| 101 | Sex | 1. Female<br>2. Male | |
| 102 | What is your marital status? | 1. Single<br>2. Married<br>3. Divorced<br>4. Widowed<br>5. Separated | |
| 103 | What is your educational level? | 1. Do not write or read<br>2. Elementary<br>3. Secondary<br>4. College diploma or above | |
| 104 | What is your occupation? | 1. Government employee<br>2. Private<br>3. Retired<br>4. Unemployed<br>5. Student<br>6. Farmer | |
| 105 | What is your average monthly income? | ----------------------- in ETB | |
| 106 | What is your religion? | 1. Orthodox Christian<br>2. Muslim<br>3. Catholic<br>4. Protestant<br>5. Other specify ----------- | |

| 2. Information on chronic HIV care follow up | | | |
|---|---|---|---|
| No. | Question | Response | Code |

| | | | | |
|---|---|---|---|---|
| 201 | | What was the point of entry for HIV testing? | 1. VCT<br>2. PITC<br>3. Medical check up<br>4. Other specify-------------------------------------- | |
| 202 | | Have you disclosed your HIV status to your partner at the time of ART initiation? | 1. Yes<br>2. No<br>3. Not applicable | |
| 203 | | What was the HIV status of your partner at the time of ART initiation? | 1. Positive<br>2. Negative<br>3. Unknown<br>4. Not applicable | |
| 204 | | Have you ever taken any ARV drug before starting HAART? | 1. Yes<br>2. No | |
| 205 | | If yes to # 204, how many ARV drugs did you take at a time? | 1. One<br>2. Two<br>3. Three | |
| 206 | | If yes to # 204, What was the reason? | 1. PMTCT<br>2. Post exposure prophylaxis<br>3. Unspecified | |
| 207 | | Have you ever been told to have Tuberculosis after starting HAART? | 1. Yes<br>2. No | |
| 208 | | Have you ever interrupted taking your medications?(for cases before switch to second-line ART) | 1. Yes<br>2. No | |
| 209 | | If yes to # 208, for how many consecutive days? | ----------------- days | |
| 210 | | If yes to # 208, what was the reason?<br><br>(Multiple answers are possible) | 1. Drug stock out at the pharmacy<br>2. Due to illness<br>3. Due to side effect<br>4. Did not have food to eat when taking pills<br>5. Away from home<br>6. Forgot<br>7. Pills were lost<br>8. Other specify | |

| | | | |
|---|---|---|---|
| 211 | Have you ever missed your clinic appointment before? | 1. Yes<br>2. No | |
| 212 | If yes to # 211, what was the reason?<br><br>(Multiple answers are possible) | 1. Distance<br>2. Transport shortage<br>3. Financial constraints<br>4. Illness<br>5. Other specify--------- | |

**Part Two: Medical record review of HIV care intake form and follow up form**

| No. | Required variables | Findings |
|---|---|---|
| 1 | Unique ART number | |
| 2 | Age at enrollment in years | |
| 3 | Date first-line ART started (dd/mm/yyyy) in EC | |
| 4 | WHO clinical stage | |
| | • At baseline | |
| | • At 6 months on ART | |
| | • At 12 months on ART | |
| | • At 24 months on ART | |
| 5 | Functional status at ART initiation | 1. Working<br>2. Ambulatory<br>3. Bedridden<br>4. Not documented |
| 6 | $CD_4$ cell counts | |
| | • At baseline | |
| | • AT 6 months on ART | |
| | • At 12 months on ART | |
| | • At 24 months on ART | |
| 7 | Peak CD4+ value (for patients on second-line regimen the peak before switch) | |
| 8 | Month on ART for peak $CD_4$ value | |
| 9 | Weight measurements | |
| | • At baseline | |
| | • At 6 months on ART | |
| | • At 12 months on ART | |
| 10 | Baseline BMI ($kg/m^{2}$) | |
| 11 | Baseline hemoglobin (gm/dl) or Hematocrit (%) | |
| 12 | Initial ARV regimen | 1. D4t + 3TC+ NVP (1a)<br>2. D4t + 3TC +EFV (1b)<br>3. AZT + 3TC + NVP (1c)<br>4. AZT + 3TC + EFV (1d)<br>5. Other specify |
| 13 | Number of drug substitutions made | 1. None<br>2. Once<br>3. Twice or more |
| 14 | Reason for drug substitution (multiple answers are possible) | 1. Side effect<br>2. Pregnancy<br>3. Risk of pregnancy<br>4. Due to new Tuberculosis<br>5. New drug available<br>6. Drug stock out<br>7. Other specify |

| | | |
|---|---|---|
| 15 | Adherence status (of the last 3 visits prior to second-line initiation for cases or prior to the current visit for controls) | 1. Good<br>2. Fair<br>3. Poor |
| 16 | Keeping appointment schedule (use the same period as for the adherence assessment) | 1. Came on time<br>2. Came late<br>3. Came early |
| 17 | Diagnosis of recurrent pneumonia ( > 2 episodes) | 1. Yes 2. No |
| **18** | **Diagnosed to have antiretroviral treatment failure** | **1. Yes 2. No** |
| 19 | If yes to #18, month on ART at the time of second-line regimen initiation | |
| 20 | If no to #18, current month on ART | |

**Name and signature of the data collector ---------------------------------------------**
